# Relationship Between Depression, Anxiety, and Antihypertensive Medication Use Among Adults With Hypertension: A Descriptive Analysis Using the National Health and Nutrition Examination Survey (NHANES) 2013-2018

**DOI:** 10.7759/cureus.104788

**Published:** 2026-03-06

**Authors:** Hillary C Ugwu, Akinyele Oladimeji, Akintunde C Akinboboye, Oyindamola D Duyilemi, Tariro Mahonde, Mofoluwakemi Johnson, Roseline Igbadumhe, Joseph E Igetei

**Affiliations:** 1 Intensive Care Unit, Prince Mutaib Bin Abdulaziz Hospital, Sakaka, SAU; 2 Family Medicine, Obafemi Awolowo University, Ile-Ife, NGA; 3 Emergency Department, University of Medical Sciences Teaching Hospital, Ondo City, NGA; 4 Psychiatry, All Saints University School of Medicine, Roseau, DMA; 5 Emergency Department, Queen Elizabeth Central Hospital, Blantyre, MWI; 6 Clinical Psychology, Walden University, Minneapolis, USA; 7 Psychology, Alaska Native Medical Center, Anchorage, USA; 8 General Medicine, International University of the Health Sciences, Basseterre, KNA

**Keywords:** anxiety, blood pressure control, depression, hypertension, medication adherence, nhanes

## Abstract

Background: Hypertension continues to be a major public health burden, with poor blood pressure (BP) control often linked to inadequate antihypertensive medication use. Psychological factors such as depression and anxiety may further complicate treatment outcomes.

Objective: The study aimed to examine the associations between depressive symptoms and anxiety-related symptoms with antihypertensive medication use and BP control among United States (U.S.) adults with hypertension.

Methods: Data from the National Health and Nutrition Examination Survey (NHANES) 2013-2018 were analyzed. Adults aged ≥18 years with diagnosed hypertension were included. Depressive symptoms were assessed using the nine-item Patient Health Questionnaire (PHQ-9). Anxiety symptoms were defined using NHANES mental health questionnaire items that assessed the frequency of feeling nervous, anxious, or unable to control worrying over the past two weeks. Antihypertensive medication use was defined as self-reported current use of prescription medication for high BP at the time of the NHANES interview, and BP control was determined using measured values. Survey-weighted logistic regression models were used to estimate adjusted ORs and 95% CIs. The unweighted analytic sample included 3,377 hypertensive adults after excluding participants with missing data on depression score and anxiety symptoms.

Results: Among 46,509,348 hypertensive adults, 39,247,780 (84%) reported taking antihypertensive medication. Depression and anxiety were not significantly associated with antihypertensive medication use or BP control after full adjustment. However, antihypertensive medication use was strongly related to BP control (OR = 1.72, 95% CI: 1.25-2.35).

Conclusion: Psychological symptoms were not independently associated with antihypertensive medication use after adjustment for demographic and clinical factors, but consistent medication use significantly improved hypertension outcomes. Integrating behavioral and clinical strategies may strengthen hypertension management in the general population.

## Introduction

Hypertension remains one of the most significant public health challenges both in the United States (U.S.) and globally, affecting approximately half of all adults and contributing substantially to cardiovascular morbidity and mortality [1]. Although effective antihypertensive drugs exist, blood pressure (BP) control rates remain suboptimal, and less than half of all patients who have hypertension that is diagnosed attain the target levels [2,3]. Psychological distress, especially depression and anxiety, has become such a critical yet insufficiently considered determinant of antihypertensive medication use and cardiovascular outcomes, among other factors leading to poor BP control [4,5].

Depression and anxiety are prevalent comorbidities in adults with hypertension, and it has been established that up to 30% of hypertensive patients show clinically significant depression or anxiety symptoms [6]. These mental disorders may have an impact on various areas of hypertension care, which include compliance with prescribed drugs, participation in lifestyle change, and regular attendance at healthcare follow-up [7,8]. Depression has been found to affect motivation, concentration, and self-efficacy, which are critical in sustaining compliance to complicated treatment regimens [9]. Equally, anxiety can increase the physiological effects of being stressed, such as sympathetic activity and high cortisol levels, which worsen the dysregulation of BP [10]. Combined, these psychological considerations can lead to a vicious cycle whereby mental stress exacerbates compliance and cardiovascular management, which then intensifies psychological stress [11,12].

The key process to achieving optimal control of hypertension is antihypertensive medication use [13]. Non-antihypertensive medication use, which can be both deliberate and unintended, is estimated to account for approximately 30-50 of the treatment failures and 10% of cardiovascular-related hospitalization in hypertensive adults [14]. Depression has been found to be associated with evidence that depressive symptomatic individuals have less tendency to refill, continue medications, and regularly self-monitor BP [15]. Although anxiety can be accompanied by an increased health vigilance, it can also lead to uneven use of medications caused by the fear of side effects or the perceived ineffectiveness of the treatment [16]. Such trends indicate the need to learn more about the psychological aspects of the medication adherence phenomenon, especially in groups where there is a chronic condition such as hypertension [17].

Prior research has reported an association between depression, anxiety, and poorer hypertension outcomes, although much of this evidence comes from clinical or disease-specific cohorts rather than population-based samples [18]. Moreover, very limited studies have investigated this correlation in the U.S. population using objective, nationally representative data that combine psychological screening, medication adherence, and measured BP results [19]. Addressing this gap is essential for understanding the real-world impact of psychological distress on hypertension management.

The present study utilized data from the National Health and Nutrition Examination Survey (NHANES) 2013-2018 to examine the connection between depressive and anxiety symptoms and medication compliance in adults diagnosed with hypertension. NHANES provides data to examine these relationships because it gathers detailed information on physical health, mental health indicators, medication use, and clinical outcomes in a representative sample of adults in the U.S. [20,21]. Through analysis of the associations, the study sought to explain the role of psychological distress in poor BP control in the real world. The findings contribute to the growing evidence base supporting integrated care approaches that address both mental health and chronic disease management to improve cardiovascular health outcomes.

## Materials and methods

Study design and data source

This study employed a cross-sectional design using data from NHANES spanning years 2013 to 2018 [21]. NHANES is a nationally representative survey of the civilian, noninstitutionalized U.S. population conducted by the National Center for Health Statistics (NCHS). The survey utilizes a complex, multistage probability sampling design that combines interviews, physical examinations, and laboratory assessments. Detailed survey protocols, consent procedures, and analytic guidelines are publicly available through the NCHS website.

Study population

This study was limited to adults aged 18 years and older who self-reported a physician diagnosis of hypertension (BPQ020, which asks whether participants had ever been told by a doctor or other health professional that they had hypertension or high BP), thereby excluding individuals with undiagnosed hypertension. Because diagnosis is influenced by healthcare access, utilization patterns, and health-seeking behavior, the findings may not be generalizable to all hypertensive adults. Moreover, mental health status may itself affect healthcare engagement, potentially introducing selection bias into the analytic sample. Participants missing key exposure, outcome, or design variables were excluded following data preprocessing and variable harmonization across survey cycles. The final unweighted analytic sample consisted of 3,377 participants, representing approximately 46,509,348 U.S. adults with hypertension when survey weights were applied.

Variables and measures

The primary exposures of interest were depression severity and anxiety symptoms. Depression was assessed using the nine-item Patient Health Questionnaire (PHQ-9) and categorized as none/mild (0-4), mild (5-9), and moderate-severe (≥10) using clinically validated thresholds [22].

Depressive symptoms were assessed using PHQ-9 administered within NHANES. Anxiety-related symptoms were approximated using selected mental health questionnaire items assessing frequency of feeling nervous or unable to control worrying over the past two weeks. NHANES 2013-2018 did not include a validated Generalized Anxiety Disorder-7 (GAD-7) module; therefore, these items were used as proxy indicators of anxiety-related symptomatology rather than a diagnostic measure. Participants reporting symptoms occurring more than half the days or nearly every day were categorized as having elevated anxiety-related symptoms.

The main outcomes were antihypertensive medication use and BP control. Antihypertensive medication use was determined based on self-reported use of prescribed antihypertensive medications. BP control was defined as a mean systolic BP <140 mmHg and a diastolic BP <90 mmHg based on the average of up to three valid readings, consistent with prevailing clinical standards during most of the 2013-2018 study period and commonly used definitions in population-based surveillance analyses.

Covariates included age (in years), gender (male/female), race/ethnicity (Mexican American, Other Hispanic, Non-Hispanic White and Non-Hispanic Black), body mass index (BMI) (in kg/m²), educational attainment (less than high school, high school/general education development (GED), college or higher), and the income-to-poverty ratio, a continuous indicator of socioeconomic status. All variables were harmonized and recorded following NHANES analytic conventions.

Statistical analysis

Descriptive statistics were computed to characterize the study population. Continuous variables were summarized as weighted means and SD, and categorical variables as weighted percentages.

Because this analysis uses complex survey design data to produce nationally representative estimates, survey-design-based F-tests were used to compare groups for categorical variables, and survey-adjusted t-tests were used for continuous variables.

Weighted logistic regression models were fitted to estimate the associations between depression, anxiety, antihypertensive medication use, and BP control. Three models were estimated for each outcome and fully adjusted for socioeconomic and clinical factors. Variance inflation factor (VIF) analysis indicated no significant multicollinearity (mean VIF = 1.65, range 1.02-2.81). All analyses were performed using Stata version 18 (StataCorp LLC, College Station, USA) [23].

Handling of missing data

Missing data were addressed using complete-case analysis. Although multiple imputation can be applied in complex survey datasets, we elected not to use imputation given the structure of missingness and to maintain consistency with standard NHANES analytic practices. Sex and age were complete for all participants. The proportion of missing data was 10.3% for the income-to-poverty ratio, 12.3% for BMI, 31.6% for systolic and diastolic BP, 41.4% for PHQ-9 depression score, 46.1% for anxiety-related symptom items, and 80.0% for antihypertensive medication use. The high proportion of missing data for medication use reflects NHANES skip patterns, as detailed medication questions are only administered to participants who report current prescription use; this represents structural missingness rather than item nonresponse. Analyses were therefore restricted to participants with complete data on the exposure, outcome, and covariates included in each regression model. The potential for selection bias related to complete-case analysis is acknowledged in the limitations.

Ethical considerations

The NHANES protocol was approved by the NCHS Research Ethics Review Board, and written informed consent was obtained from all participants. Because this analysis used publicly available, de-identified data, additional institutional review board approval was not required. All analyses were conducted in compliance with NHANES analytic guidelines and ethical standards for secondary data research.

## Results

Table 1 presents the weighted demographic, clinical, and psychosocial characteristics of U.S. adults with diagnosed hypertension, stratified by antihypertensive medication use status.

**Table 1:** Weighted characteristics of adults with diagnosed hypertension by antihypertensive medication use, NHANES 2013-2018 (unweighted N = 3,377) Values represent survey-weighted population estimates. Percentages represent row percentages. Group comparisons for categorical variables were conducted using Rao-Scott adjusted chi-square tests (reported as F-statistics), while survey-adjusted t-tests were used for continuous variables, accounting for the complex survey design. Analyses were conducted using NHANES 2013-2018 data [21] in Stata version 18 [23]. NHANES: National Health and Nutrition Examination Survey; PHQ-9: nine-item Patient Health Questionnaire; BMI: body mass index; BP: blood pressure; GED: general education development

Characteristic	Not taking medications (N = 7,261,568)	Taking medications (N = 39,247,780)	F-test/t-test	p-value
PHQ-9 total (mean ± SD)	4.03 ± 4.79	3.67 ± 4.63	t = 0.95	0.349
BMI in kg/m² (mean ± SD)	32.01 ± 7.40	31.95 ± 7.23	t = 0.11	0.912
Age in years (mean ± SD)	50.94 ± 13.51	62.13 ± 12.61	t = -12.52	<0.001
Income-to-poverty ratio (mean ± SD)	2.75 ± 1.51	3.04 ± 1.63	t = -2.42	0.020
Mean systolic BP in mmHg (mean ± SD)	133.83 ± 18.13	131.14 ± 18.46	t= 2.51	0.016
Mean diastolic BP in mmHg (mean ± SD)	76.25 ± 12.75	70.36 ± 13.84	t = 9.11	<0.001
Gender (n, %)	-	-	F = 1.56	0.218
Male	3,762,304 (17%)	18,664,035 (83%)	-	-
Female	3,499,264 (15%)	20,583,745 (85%)	-	-
Education level (n, %)	-	-	F = 0.112	0.856
Less than high school	987,433 (15%)	5,608,459 (85%)	-	-
High school graduate/GED	1,890,199 (15%)	10,499,424 (85%)	-	-
College+	4,383,936 (16%)	23,139,897 (84%)	-	-
Depression (n, %)	-	-	F = 0.339	0.704
None/mild	5,014,299 (15%)	27,862,276 (85%)	-	-
Mild	1,447,910 (17%)	6,990,336 (83%)	-	-
Moderate-severe	799,359 (15%)	4,395,169 (85%)	-	-
Anxiety (n, %)	-	-	F = 0.625	0.557
None/mild	5,406,377 (15%)	29,850,984 (85%)	-	-
Moderate-severe	1,855,191 (16%)	9,396,797 (84%)	-	-
BP control (n, %)	-	-	F = 7.460	0.009
Uncontrolled	4,656,996 (18%)	21,362,509 (82%)	-	-
Controlled	2,604,572 (13%)	17,885,272 (87%)	-	-
Race/ethnicity (n, %)	-	-	F = 4.342	0.013
Mexican American	615,782 (23%)	2,012,199 (77%)	-	-
Other Hispanic	303,320 (15%)	1,766,388 (85%)	-	-
Non-Hispanic White	5,419,166 (15%)	29,873,234 (85%)	-	-
Non-Hispanic Black	923,300 (14%)	5,595,960 (86%)	-	-

From Table 1, it is evident that among an estimated 46,509,348 hypertensive adults, 39,247,780 (84%) reported taking BP medication, while 7,261,568 (16%) did not. Participants currently taking antihypertensive medication were older (62.1 ± 12.6 years) than those not currently taking antihypertensive medication (50.9 ± 13.5 years, p < 0.001). The groups had similar BMI (31.9 ± 7.2 kg/m² vs. 32.0 ± 7.4 kg/m²) and depression severity (PHQ-9 mean 3.7 ± 4.6 vs. 4.0 ± 4.8). The income-to-poverty ratio was slightly higher among adherent adults (3.0 ± 1.6 vs. 2.8 ± 1.5, p = 0.02). Mean systolic and diastolic pressures were lower among those taking medication (131.1 ± 18.5 mmHg and 70.4 ± 13.8 mmHg) than among those not taking it (133.8 ± 18.1 mmHg and 76.3 ± 12.8 mmHg, both p < 0.05), indicating better BP control. Antihypertensive medication use did not significantly differ by gender or education level, and depressive or anxiety symptom categories showed no significant associations with antihypertensive medication use. However, BP control and race/ethnicity varied significantly (p = 0.009 and p = 0.013, respectively), with a lower proportion of uncontrolled hypertension among adherent adults and small differences observed across racial/ethnic subgroups.

Table 2 presents results from the fully adjusted logistic regression examining the association between depression, anxiety symptoms, and antihypertensive medication use among U.S. adults with hypertension.

**Table 2:** Survey-weighted logistic regression analysis with antihypertensive medication use as the outcome variable among adults with hypertension, NHANES 2013-2018 ORs and 95% CIs were estimated using survey-weighted models to account for the complex NHANES design. The reference categories for depression, anxiety, gender, and race/ethnicity were “none/mild,” “none/mild,” “male,” and “Mexican American,” respectively. Analyses were adjusted for age, gender, race/ethnicity, education, BMI, and income-to-poverty ratio. Outcome variable: antihypertensive medication use (taking medication/not taking medication), defined as self-reported use of prescribed antihypertensive drugs. Analyses were conducted using NHANES 2013-2018 data [21] in Stata version 18 [23]. NHANES: National Health and Nutrition Examination Survey; BMI: body mass index; GED: general education development

Predictor	OR (95% CI)	p-value
Depression category	-	-
Mild vs. none/mild	0.91 (0.59-1.41)	0.676
Moderate-severe vs. none/mild	1.25 (0.66-2.39)	0.480
Anxiety symptoms	-	-
Moderate-severe vs. none/mild	0.92 (0.58-1.47)	0.730
Age in years	1.07 (1.06-1.08)	<0.001
Gender	-	-
Female vs. male	1.02 (0.77-1.34)	0.907
Race/ethnicity	-	-
Other Hispanic vs. Mexican American	1.69 (1.08-2.64)	0.024
Non-Hispanic White vs. Mexican American	1.12 (0.80-1.56)	0.512
Non-Hispanic Black vs. Mexican American	1.83 (1.34-2.49)	<0.001
BMI in kg/m²	1.03 (1.00-1.05)	0.017
Income-to-poverty ratio	1.15 (1.04-1.27)	0.005
Education level	-	-
High school graduate/GED vs. less than high school	0.90 (0.61-1.31)	0.572
College+ vs. less than high school	0.75 (0.50-1.13)	0.165

After adjusting for potential confounders, neither depression nor anxiety symptoms were significantly associated with antihypertensive medication adherence. Compared with adults reporting no or mild depressive symptoms, those with mild depression (OR = 0.91, 95% CI: 0.59-1.41) or moderate-severe depression (OR = 1.25, 95% CI: 0.66-2.39) had similar odds of medication use. Similarly, moderate-severe anxiety was not associated with adherence (OR = 0.92, 95% CI: 0.58-1.47). Age (OR = 1.07, 95% CI: 1.06-1.08) and higher income-to-poverty ratio (OR = 1.15, 95% CI: 1.04-1.27) were positively associated with antihypertensive medication use, while BMI also showed a modest positive relationship (OR = 1.03, 95% CI: 1.00-1.05). Compared with Mexican American adults, Non-Hispanic Black adults were significantly more likely to report medication use (OR = 1.83, 95% CI: 1.34-2.49). No significant differences were observed by gender or education level.

Table 3 summarizes the fully adjusted survey-weighted logistic regression results assessing the relationship between depression, anxiety, antihypertensive medication use, and BP control among hypertensive adults in the U.S. The analysis controlled for age, sex, race/ethnicity, education level, BMI, and income-to-poverty ratio.

**Table 3:** Survey-weighted logistic regression analysis with BP control as the outcome variable among adults with hypertension, NHANES 2013-2018 ORs and 95% CIs were estimated using survey-weighted logistic regression to account for the complex NHANES sampling design. The unweighted analytic sample included 3,377 participants. For the medication model, the outcome was current antihypertensive medication use (taking medication vs. not taking medication), defined as self-reported use of prescribed antihypertensive drugs. For the BP control model, the outcome was controlled BP (systolic <140 mmHg and diastolic <90 mmHg) versus uncontrolled. Analyses were conducted using NHANES 2013-2018 data [21] in Stata version 18 [23]. NHANES: National Health and Nutrition Examination Survey; BMI: body mass index; BP: blood pressure; GED: general education development

Predictor	OR (95% CI)	p-value
Depression category	-	-
Mild vs. none/mild	1.06 (0.78-1.44)	0.704
Moderate-severe vs. none/mild	1.07 (0.76-1.51)	0.689
Anxiety symptoms	-	-
Moderate-severe vs. none/mild	0.87 (0.65-1.18)	0.376
Current antihypertensive medication use	-	-
Taking medication vs. not taking medication	1.72 (1.25-2.35)	0.001
Age in years	0.99 (0.98-1.00)	0.020
Gender	-	-
Female vs. male	1.14 (0.95-1.37)	0.148
Race/ethnicity	-	-
Other Hispanic vs. Mexican American	0.62 (0.40-0.95)	0.029
Non-Hispanic White vs. Mexican American	0.91 (0.73-1.14)	0.423
Non-Hispanic Black vs. Mexican American	0.57 (0.43-0.74)	<0.001
BMI in kg/m²	1.00 (0.98-1.01)	0.744
Income-to-poverty ratio	1.06 (0.98-1.14)	0.130
Education level	-	-
High school graduate/GED vs. less than high school	1.13 (0.87-1.46)	0.347
College+ vs. less than high school	1.22 (1.00-1.50)	0.050

The fully adjusted model in Table 3 reveals that depression and anxiety symptoms were not significantly associated with BP control. Participants with mild (OR = 1.06, 95% CI: 0.78-1.44) or moderate-severe depression (OR = 1.07, 95% CI: 0.76-1.51) had similar odds of achieving BP control compared with those with none or mild symptoms. Likewise, moderate-severe anxiety was not significantly related to BP control (OR = 0.87, 95% CI: 0.65-1.18). However, individuals who reported taking antihypertensive medications were significantly more likely to have controlled BP (OR = 1.72, 95% CI: 1.25-2.35, p = 0.001). Older age was modestly associated with lower odds of control (OR = 0.99, 95% CI: 0.98-1.00), while college education showed a borderline positive association (OR = 1.22, 95% CI: 1.00-1.50, p = 0.050). Compared with Mexican American adults, Non-Hispanic Black adults were less likely to have controlled BP (OR = 0.57, 95% CI: 0.43-0.74).

## Discussion

This study evaluated the associations between depressive symptoms, anxiety symptoms, antihypertensive medication use, and BP control among U.S. adults with diagnosed hypertension using nationally representative NHANES data from 2013 to 2018. Medication use was reported by a substantial majority of hypertensive adults, and consistent adherence was strongly associated with BP control. In contrast, depressive and anxiety symptoms were not independently associated with either antihypertensive medication use or BP control after adjustment for demographic, socioeconomic, and clinical factors. These findings suggest that in treated, population-based samples, psychological distress alone may not be a primary determinant of measurable hypertension outcomes.

Prior literature has consistently linked depression and anxiety with poorer hypertension outcomes, largely through impaired self-care behaviors and reduced treatment persistence [4,7,9]. Meta-analyses and clinical cohort studies have reported that depressive symptoms interfere with motivation, executive function, and routine health behaviors required for chronic disease management [6,9]. However, much of this evidence originates from clinical populations with higher symptom severity and more complex treatment regimens. In contrast, population-based data such as NHANES capture a broader range of symptom burden, where most individuals report mild or moderate distress rather than major depressive or anxiety disorders. In such contexts, habitual medication taking and engagement with primary care may remain relatively stable, potentially explaining the absence of a significant association between psychological symptoms and adherence in this analysis, consistent with prior NHANES-based findings [19,20].

Psychological distress has well-described biological links to hypertension through neuroendocrine and autonomic pathways. Depression and anxiety activate the hypothalamic-pituitary-adrenal axis, increasing cortisol secretion, which contributes to endothelial dysfunction, sodium retention, and elevated vascular resistance [10]. Simultaneously, heightened sympathetic nervous system activity and reduced parasympathetic tone promote vasoconstriction, impaired baroreflex sensitivity, and increased arterial stiffness [10,11]. These mechanisms provide a biologically plausible pathway connecting psychological distress to BP dysregulation. However, in populations receiving antihypertensive therapy, the clinical manifestation of these pathways may be attenuated. Antihypertensive agents may influence sympathetic activity and vascular function, which have been implicated in stress-related cardiovascular pathways; however, these mechanisms were not directly evaluated in the present analysis [9,15].

The absence of a significant association between anxiety symptoms and BP control aligns with growing evidence that anxiety-related effects on cardiovascular outcomes are variable and context dependent. While severe anxiety may exacerbate autonomic imbalance and vascular reactivity, milder anxiety symptoms may increase health vigilance and treatment engagement, potentially offsetting adverse physiological effects [5,10,16]. At the population level, these opposing influences may result in neutral associations once treatment status and socioeconomic factors are accounted for, as observed in this analysis.

Antihypertensive medication use emerged as the strongest determinant of BP control. Antihypertensive therapies directly modulate the core biological systems sustaining hypertension, including suppression of the renin-angiotensin-aldosterone system, reduction of peripheral vascular resistance, and regulation of sodium and water balance [8,13]. Sustained adherence allows these mechanisms to operate consistently, producing stable BP reduction. Conversely, participants not currently taking antihypertensive medication lead to unopposed neurohumoral activation, increased vascular tone, and volume expansion, a pattern frequently misclassified as resistant hypertension rather than insufficient pharmacologic exposure [14]. These findings reinforce that biological control of hypertension is primarily driven by consistent medication use rather than psychosocial influences acting independently.

Sociodemographic differences observed in antihypertensive medication use and BP control further support the role of structural determinants. Older age and higher income were associated with greater medication use, while racial and ethnic disparities persisted in BP control despite adjustment for adherence and socioeconomic status. These findings align with prior work demonstrating that access to care, treatment intensity, and cumulative cardiovascular risk contribute to persistent disparities in hypertension outcomes [2,3,18].

Although depression and anxiety were not independently associated with antihypertensive medication use or BP control, their clinical relevance remains important. Psychological distress may influence hypertension indirectly through lifestyle behaviors, healthcare utilization, and long-term cardiovascular risk accumulation [11,12,18]. Population studies indicate that depression more strongly predicts cardiovascular morbidity and mortality than short-term medication persistence, suggesting effects that operate through prolonged biological and behavioral pathways rather than immediate non-use of antihypertensive medication [18]. Routine mental health screening within hypertension care settings may therefore remain valuable for comprehensive risk management, even when medication adherence appears adequate.

Strengths and limitations of the study

This study benefits from the use of standardized psychological screening tools, objectively measured BP, and nationally representative data. Adjustment for key demographic and socioeconomic factors strengthened internal validity. Limitations include reliance on self-reported medication use, which may introduce reporting bias, and substantial missingness in psychological variables inherent to the NHANES survey design. Anxiety was not assessed using a validated diagnostic instrument such as the GAD-7. Instead, proxy symptom items were used due to limitations of available NHANES variables. This may result in misclassification and limit the interpretation of findings related to anxiety. The cross-sectional design also limits causal inference.

Future research should prioritize longitudinal designs to clarify temporal relationships between psychological distress, medication persistence, and BP trajectories. Incorporation of objective adherence measures and validated anxiety instruments would enhance precision. Integrated care models that simultaneously address pharmacologic antihypertensive medication use and mental health support may offer the greatest opportunity to improve long-term hypertension outcomes at the population level [12].

## Conclusions

This study highlights the relationship between psychological symptoms, antihypertensive medication use, and BP control among U.S. adults with hypertension using nationally representative data. Depressive and anxiety symptoms were not independently associated with antihypertensive medication use or BP control after accounting for clinical and socioeconomic factors. In contrast, consistent medication adherence remained strongly linked to effective BP management. These findings reinforce the central role of sustained pharmacologic treatment in hypertension care within the general population. Psychological distress remains clinically relevant but may exert its effects through longer-term behavioral or biological pathways rather than immediate treatment behaviors. Future research should apply longitudinal designs and objective antihypertensive medication use measures to clarify temporal relationships and to inform integrated care strategies that support both cardiovascular and mental health management.
